# Effect of Inert Gas Cover on the Static and Fatigue Behavior of AA6061-T6 Aluminum Alloy Friction Stir Spot Lap-Shear Welds

**DOI:** 10.3390/ma18020256

**Published:** 2025-01-09

**Authors:** Amir Alkhafaji, Daniel Camas, Hayder Al-Asadi

**Affiliations:** 1 Department of Civil and Materials Engineering, University of Malaga, E-2971 Malaga, Spain; dcp@uma.es; 2 Department of Mechanical Engineering, Faculty of Engineering, University of Isfahan, Isfahan 817467344, Iran; h.k.alasadi@gmail.com

**Keywords:** FSSW, inert gas cover, fatigue behavior, oxide layer, crack propagation, interfacial crack, bond line oxidation

## Abstract

Friction stir spot welding (FSSW) technology relies on the generation of frictional heat during the rotation of the welding tool in contact with the workpiece as well as the stirring effect of the tool pin to produce solid-state spot joints, especially for lightweight materials. Although FSSW offers significant advantages over traditional fusion welding, the oxidation of the interfacial bond line remains one of the most challenging issues, affecting the quality and strength of the joint under both static and cyclic loading conditions. In this experimental study, inert argon gas was employed to surround the joint, aiming to prevent or minimize the formation of the interfacial oxides. Two welding processes were conducted with identical welding process parameters and welding tool geometry: the conventional process and another that employs an inert gas cover. Micrographs of as-welded specimens were analyzed using a computerized optical microscope to characterize the interfacial bond lines and an energy-dispersive spectroscope (EDS) was used to quantify the interfacial oxides. Specimens from both welding conditions were tested under static and cyclic loads to investigate the static and fatigue behaviors, respectively. The fatigue tested specimens were examined under different load levels to investigate the fatigue crack behavior and the modes of failure at low-cycle and high-cycle fatigue conditions. The optical micrographs showed significant improvement in bond line morphologies (33% enlarged fully bonded area) and both static and fatigue strengths (35% reduced partially bonded area) when the inert gas cover was used. The EDS analysis revealed a maximum reduction of the interfacial oxide of 41% in the bond line achieved in the argon-surrounded joints compared to specimens of the conventional welding process. Accordingly, an improvement of 14% in the static strength was reached, along with 60% and 26% in the fatigue strengths at low- and high-cycle fatigue conditions, respectively.

## 1. Introduction

**Figure 1:**
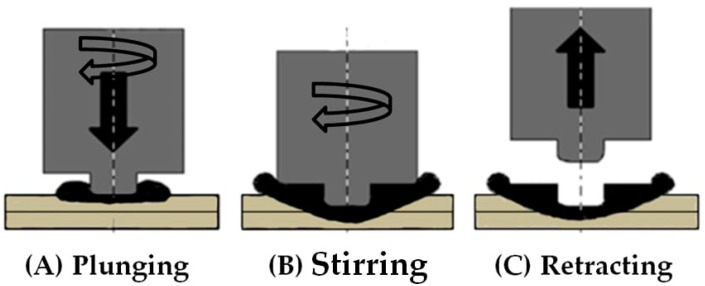
Schematic illustration of the FSSW process.

To achieve more efficient fuel consumption and reduce carbon dioxide emissions, lightweight materials such as aluminum and magnesium alloys have become increasingly attractive to researchers, engineers, and designers as promising structural materials for the automotive industry [1]. Friction stir spot welding (FSSW) has been developed as a novel variant of friction stir welding (FSW) to replace resistance spot welding (RSW) for aluminum [2]. As a solid-state welding technique, FSW/FSSW overcomes many of the defects and challenges associated with fusion welding technologies, such as excessive heat, fume, poor joints, cracking, and workpiece distortion. The initial development of FSSW was carried out by Sumitomo Light Metal Industries, Ltd. (Osaka, Japan), Mazda, Kawasaki Heavy Industries, Ltd. (Tokyo, Japan), and Norsk Hydro (Oslo, Norway) [3]. FSSW can produce lap welds in the solid state without bulk melting with a similar appearance to that of the resistance spot welds. Due to its solid-state nature and other advantages, FSSW has become appealing for structural assemblies, body manufacturing, and similar industrial applications [4,5]. FSSW is an effective method for welding light metals, such as aluminum, magnesium, copper, etc. [2,6]. As shown in Figure 1, the FSSW welding process involves three specific steps: the plunging step, where the tool is inserted to a specific depth in the workpiece; the stirring step, where the welding tool generates frictional heat and softens and incorporates together the materials of the upper and lower sheets; and the retracting step, where the tool is then withdrawn from the sample at a higher speed compared to the plunging step [7].

A typical cross-section of the FSSW weld consists of four neighboring distinct zones, namely, the stir zone (SZ) adjacent to the keyhole, the thermomechanically affected zone (TMAZ), the heat-affected zone (HAZ), and the base metal (BM). Welding process parameters and welding tool design mainly control frictional heat generation, material flow, plastic deformation, and the distribution of precipitates in the SZ. Accordingly, the morphology of the weld features in terms of the grain refinement and distribution of precipitates significantly affects the mechanical properties and failure mode of the weld joint. Higher grain refinement and a uniform distribution of precipitates in the SZ produce higher hardness and tensile strength and improved fatigue behavior [1,7,8,9]. Aluminum alloys have received significant attention and research due to their lightweight properties and moderate strength, making them promising materials for key industrial applications, including transportation, aerospace, and structural industries [7,10,11,12]. Among these, heat-treatable 6xxx aluminum alloys have been extensively studied for their moderate strength, formability, and corrosion resistance [13]. AA6061, an aluminum–magnesium–silicon (Al-Mg-Si) heat-treatable alloy, is widely used in structural components of welded assemblies due to its superior weldability compared to high-strength aluminum alloys [14,15,16]. Like other welding techniques, FSSW joints face challenges under static and cyclic loading, often leading to static yield fractures and fatigue failure, respectively [17]. Fatigue behavior is mainly influenced by the mechanical properties of the materials under cyclic loading conditions [18], as well as by the microstructure, particularly the presence of structural defects [19]. Consequently, the fatigue strength of FSSW weld joints is closely related to the welding parameters. During the welding process, frictional heat generates a thin oxide film at the interface between the two sheets, a natural outcome of the process being carried out in atmospheric conditions. As the stirring effect diminishes away from the tool pin, an array of discontinuous oxide particles disperse along the interface bond line, creating a partially metallurgical bonded area known as “kissing bonds” (KBs) [20,21].

Previous studies have frequently identified this issue in friction stir spot welding. Some researchers mentioned the phenomenon briefly within the context of their work without attempting to solve it, while others tried to partially reduce its impact by controlling the welding process parameters and the welding tool geometry.

When investigating similar and dissimilar FSSW welds, Shen et al. [1] reported that the interfacial oxide layer strongly weakens dissimilar Mg/steel welds, leading to interfacial failure. Bakavos and Prangnell [22] investigated the friction stir spot welding of thin automotive sheets and found that the oxide layer leads to the formation of kissing bonds, which may facilitate crack propagation. Klobčar et al. [4] noted that the fully metallurgical bonded area constitutes no more than 20% of the bond line and concluded that the oxide film significantly affects the FSSW weld quality, as cracks may propagate through the oxide film during loading. Shen et al. [20] correlated the interfacial oxide layer distribution to the stirring effect of the tool pin along the bond line, stating that the oxide layer breaks into discontinuous particles due to the inefficient stirring effect of the tool pin, which inhibits full metal bonding and results in a partially metallurgical bonded area. They concluded that the effective oxide milling is related to the distance from the tool pin. In a similar finding, Badarinarayan et al. [21] reported that the array of broken oxide particles forms a “hook” defect. Roa et al. [9] stated that the distribution of oxide particles at the bond line is mainly influenced by the welding tool rotational speed and tool geometry. In the same context, Wei Yuan [2] reported that the oxide film formation was detrimental to the mechanical properties of the FSSW welds in both lap-shear and cross-tension configurations. Moreover, eliminating or reducing the oxide layer generally improves the joint strength. In an attempt to reduce interfacial oxide formation, Wei Yuan reduced the tool rotational speed to decrease the frictional heat generation. This procedure may have partially reduced oxide layer formation; however, the generation of friction heat is essential in softening the workpiece material to producing friction stir spot welds. For higher weld purity and quality, traditional welding technologies have utilized different approaches to isolate the weld area: atmospheric oxygen flux and CO_2_ are used to surround molten metal in electric arc welding, while an inert gas is employed in TIG and MIG welding technologies [23,24]. In the same context, previous studies investigated using gas shielding to prevent bond line oxidation during welding. To prevent bond line and welding tool oxidation, Yaghoubi and Shirazi [25] conducted their experiments under an argon shield during the FSW of copper plates. Chen et al. [26] investigated the influence of Ar and He gas shields with four mixing ratios on the weld quality of a base metal of 5083-H111 aluminum with 20 mm thickness by ER5356 aluminum wires. They achieved a better weld formation and a significant reduction in porosity. Moreover, an improvement of 22.7% was reached in tensile strength with increasing He percentage. On the other hand, Xia et al. [27] used a ratio combination of Ar and CO_2_ as a gas shields during laser-welded–brazed dissimilar aluminum/steel lap joints. They concluded that the addition of CO_2_ enhanced fusion volume and improved the molten filler wettability. In addition, a superior tensile strength was achieved with a mixture of 50% of both Ar and CO_2_. In two separate studies, Pehkonen [28] designed and Björck et al. [29] successfully implemented a gas shield that was attached to the FSW machine of the copper canisters that are used by The Swedish Nuclear Fuel and Waste Management Company (SKB) to contain and store their nuclear residuals. They achieved oxygen-free FSW copper joints.

This study investigated an innovative experimental technique that employs a flow of inert argon gas cover that isolates the weld area from atmospheric oxygen and prevents or minimizes the oxidation of the weld bond line, in turn improving the weld quality. The morphology of the weld area was examined as they were welded and comparisons with traditional specimens were conducted to assess the effect of using this innovative technique on the static and fatigue behaviors of FSSWed AA6061-T6 lap joints.

The main goal of the study is to improve the static and fatigue strengths of the FSSW lap joints though the elimination or limitation of the bond line oxidation during welding.

## 2. Materials and Methods

**Figure 2:**
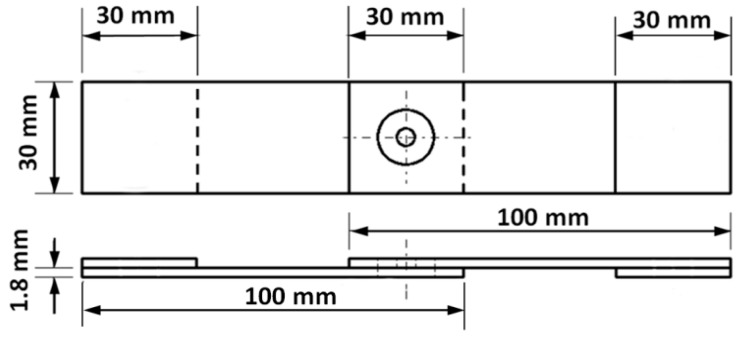
Schematic of the used FSSW lap-shear specimen.

**Figure 3:**
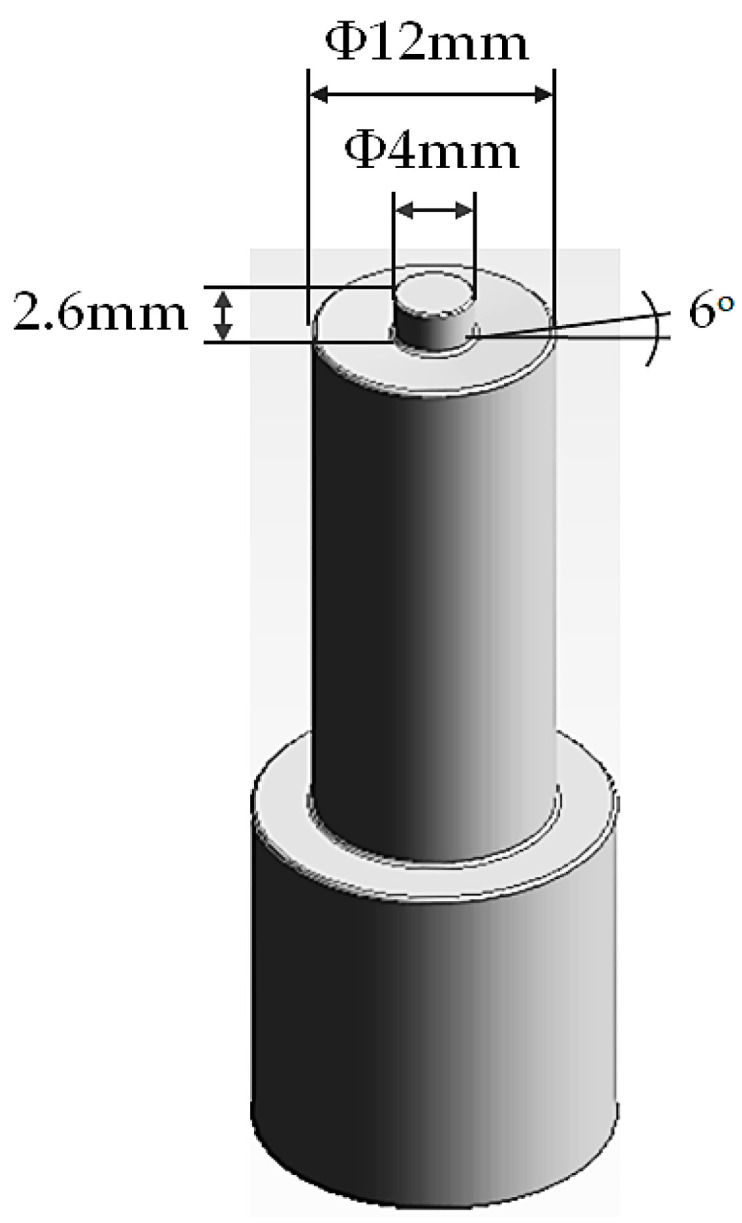
Schematic illustration of tool used for the FSSW process.

In this investigation, AA6061-T6 aluminum alloy with thickness of 1.8 mm was used to prepare the FSSW lap-shear specimens required to conduct the tensile and fatigue testing. The chemical composition (wt.%) of the selected alloy is presented in Table 1, while its mechanical properties are shown in Table 2. A pair of alloy sheets, each measuring 100 mm × 30 mm with an overlap area of 30 mm × 30 mm, was arranged in a lap configuration to prepare the specimens, as depicted in schematic Figure 2. To support the lap-shear specimens during the FSSW process and to prevent initial misalignment during testing, a pair of 30 mm × 30 mm spacers was attached to each end of the specimens [13,17,30,31,32,33,34]. The FSSW welding tool used was made from hot-work tool steel AISI H13 that had been hardened to 48 HV [7,35,36]. The tool was machined to have a shoulder diameter of 12 mm, a pin diameter of 4 mm, and a pin length of 2.6 mm. Additionally, the shoulder was concaved to an angle of 6°, as illustrated in schematic Figure 3.

To ensure accurate and comparable results, a set of pre-experimentally identified welding process parameters, namely, tool rotational speed, dwell time, plunge speed, tool retracting speed, and shoulder plunge depth, were maintained as constants throughout the entire experiment at 750 rpm, 15 s, 10 mm/min, 100 mm/min, and 0.3 mm, respectively. As a result, the effects of all these parameters on the mechanical properties, microstructure, weld nugget thickness, hook features, and geometry remained consistent throughout the experimental work. Any variations in weld quality and durability can be attributed to the effect of the use of inert gas, which was employed to surround and isolate the FSSW weld from atmospheric oxygen, thereby preventing or minimizing the formation of oxides during the welding process.

**Figure 4:**
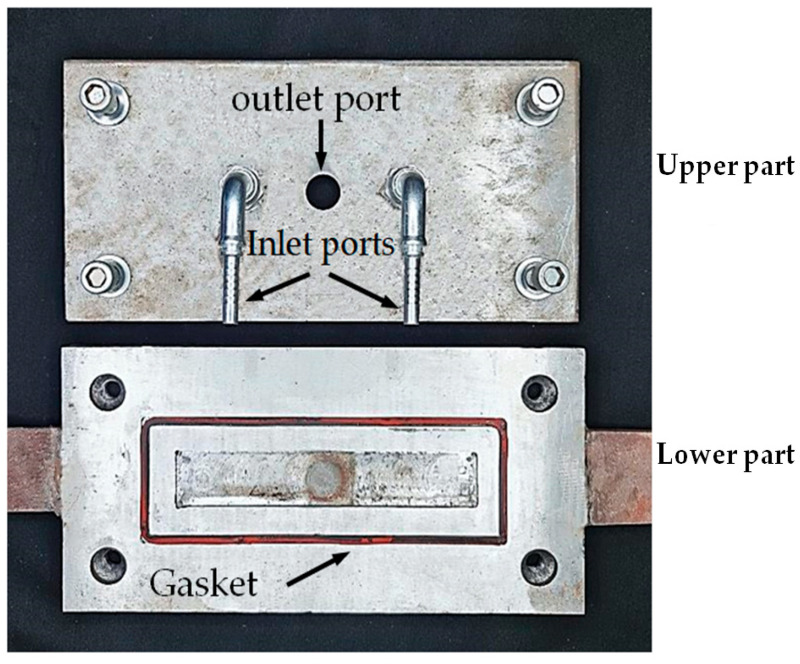
Specially designed welding mechanism, equipped with inlet paths and outlet for inert gas.

A specially designed welding mechanism, consisting of tightly connected upper and lower parts secured by four bolts, was used to contain the specimen and control the inert gas flow during the welding process. The lower part is engraved with a rectangular groove equipped with a rubber gasket that completely surrounds the lap-shear specimen and prevents any potential interfacial gas leakage. The upper part includes inlet ports connected to gas grooves, which extend from the maim inlet ports and distribute the inert gas along the FSSW lap-shear specimen to ensure that the gas surrounds, circulates, and reaches the outlet port at the center of the upper part of the mechanism, as shown in Figure 4.

**Figure 5:**
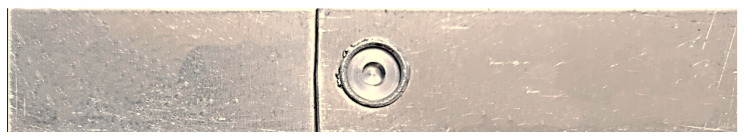
Typical performed FSSW specimen.

Before being welded, the aluminum sheets were scraped with grade-800 SiC paper and washed of possible impurities using acetone [7]. Two FSSW processes were conducted at the center of the overlap area of the lap-shear specimens for two sets of coupons. Process A was carried out in a normal atmospheric air environment, while the coupons of process B were completely surrounded by a flow of inert argon gas (Ar). After several preliminary welding processes, the argon pressure was adjusted to 1.5 bar to ensure a complete argon shield around the welding specimens, with a complete expulsion of atmospheric oxygen along with preventing any frictional heat transfer by the forced convection of argon currents. A typical FSSW weld of AA6061-T6 aluminum alloy is shown in Figure 5. Three FSSW welded specimens from both A and B sets were subjected to tensile testing at room temperature until complete separation using a QUASAR 25, Cardano al Campo (VA), Italy, tensile testing machine at a constant displacement rate of 1 mm/min. The average tensile–shear load (TSL) values were considered as references to evaluate the maximum applied load for the fatigue tests [37,38].

As-welded FSSW specimens from A and B groups were cross-sectioned by an Electro-discharge Wire Cutting Machine (EDW). They were then gradually wet-ground with different grades of silicon carbide emery paper and polished with 0.25 micron diamond paste. Next, the cross-sectioned specimens were cleaned with distilled water and alcohol, dried, and, finally, etched with Keller’s reagent solution, composed of 2.5% HNO_3_, 1.5% HCL, 1% HF, and 95% H_2_O [7,12,39].

After being calibrated by a standard sample, Vickers hardness testing device CV-400DM was kept at 300 gf and cycle time of 15 s before it was used to conduct the micro-hardness test for the cross-sectioned welds at the mid-thickness of the upper sheet with steps of 0.25 mm.

Specimens of A and B groups were tested under cyclic loading conditions for the fatigue test using the MST 809 Axial/Torsional Test System at a sinusoidal loading ratio of R = 0.1. Due to the limited testing time, frequency was controlled between 10 and 30 Hz, depending on the load level. Frequencies above 10 Hz were used to expedite the failure at lower load levels [1,37,38]. The maximum applied load for the fatigue test was considered as 80% of the TSL values with a sequential decrease of 10% for six different load levels [35,36]. Three specimens were tested at each load range to evaluate the average number of cycles to failure (N_f_) at the corresponding load range [17,35,36,37]. The test was stopped for some specimens at a life cycle of 0.75 N_f_ for some load levels to obtain partially failed specimens, which were then cross-sectioned and prepared following the same aforementioned metallurgical procedures to observe the fatigue crack initiation and propagation in conditions of low- and high-cycle fatigue [37,38].

## 3. Results and Discussion

### 3.1. Micrograph

The interfacial oxide layer, which forms due to the frictional heat during welding, is broken down by the stirring effect of the tool pin stirring into small particles and penetrate the bond line. These particles are dispersed into the stir zone, approaching the tool pin [9,21,33].

Depending on the material integration of the joint, mainly influenced by material flow and the dispersion of oxide particles, the interface of the bond line is divided into three adjacent areas from the periphery of the keyhole to the joint edge: the fully bonded area (FBA), the partially bonded area (PBA), and the unbounded area (UBA) [13,21].

**Figure 6:**
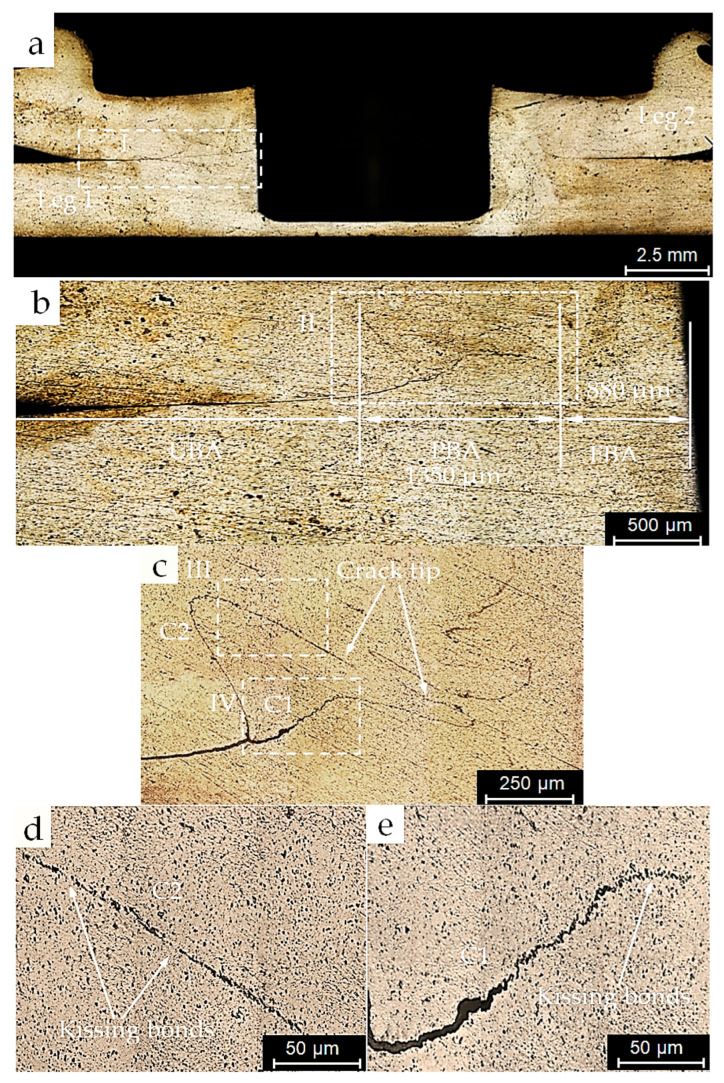
Micrograph of the cross-section of an as-welded conventional FSSW specimen performed in the surrounding atmospheric air: (**a**) joint over view; (**b**) magnified view of region I of (**a**) that represents the bond line of the joint; (**c**) magnified view of region II of (**b**) that represents the partially boned area; (**d**) magnified view of region III of (**c**) that represents the primary hook, including kissing bonds; and (**e**) magnified view of region IV of (**c**) that represents the original interfacial crack extension towards the partially bonded area.

**Figure 7:**
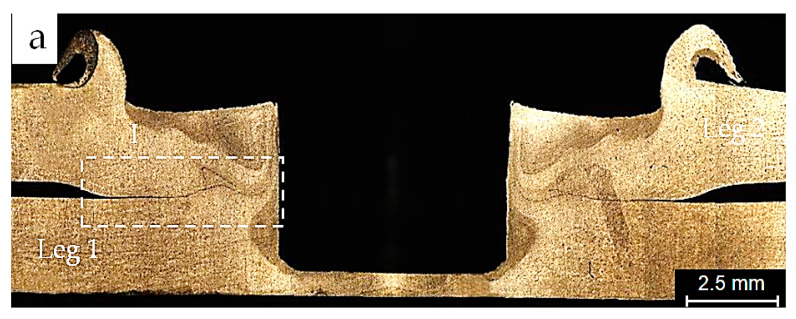

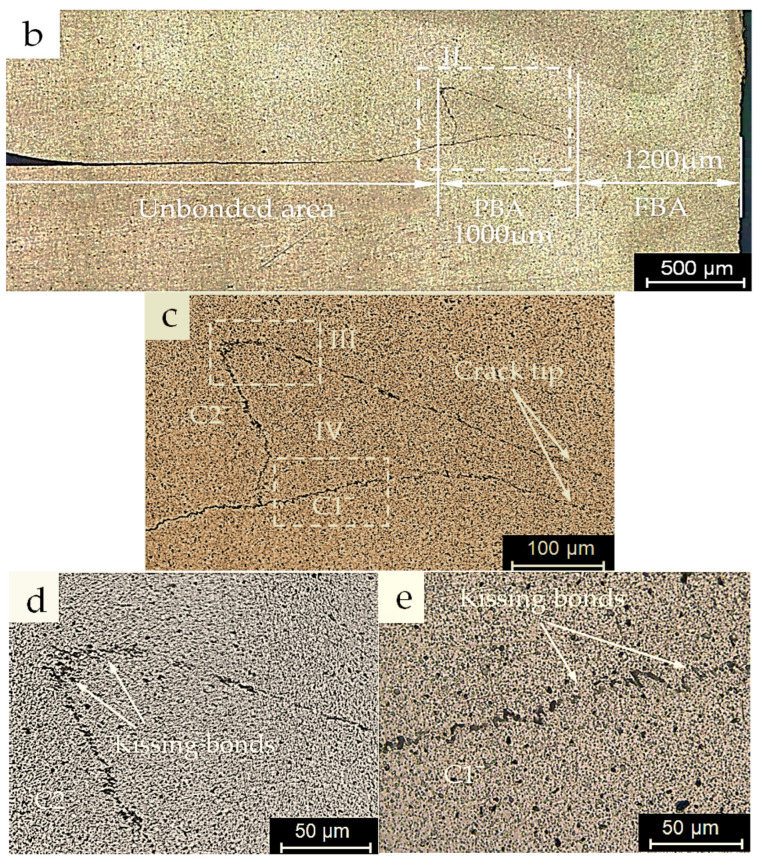
Micrograph of the cross-section of an as-welded FSSW specimen performed with an inert gas cover: (**a**) joint overview; (**b**) magnified view of region I of (**a**) that represents the bond line of the joint; (**c**) magnified view of region II of (**b**) that represents the partially boned area; (**d**) magnified view of region III of (**c**) that represents the primary hook, including kissing bonds; and (**e**) magnified view of region IV of (**c**) that represents the original interfacial crack extension towards the partially bonded area.

A Leica DM6 M computerized optical microscope (OM) was employed to examine the cross-sections along the symmetry axis of as-welded FSSW specimens from groups A and B. Figure 6 and Figure 7 show optical micrographs of the cross-sections of the bond lines of specimens from A and B processes, respectively. Figure 6a and Figure 7a show overviews of the specimens’ cross-sections, including the cylindrical probe hole, the concave shoulder indentation, and the sheet interfaces of specimens of processes A and B respectively. Figure 6b and Figure 7b represent close-up views of regions marked I in Figure 6a and Figure 7a, respectively. These figures display the bond lines, including the UBA, PBA, and FBA of each specimen. It is evident that the shape of the hook cracks and the sizes of the bond line regions differ slightly between the two specimens. The FBAs were 880 µm and 1200 µm, while the PBAs were 1350 µm and 1280 µm for the specimens from sets A and B, respectively. Moreover, the bond line of the specimen from process B, which was welded under the argon cover, appeared finer and purer compared to that of process A, which was welded in the natural ambient environment. Magnified views of regions marked II in the previous figures are shown in Figure 6c and Figure 7c, showing two cracks and their tips extending through the PBAs of the specimens from both welding processes.

**Figure 8:**
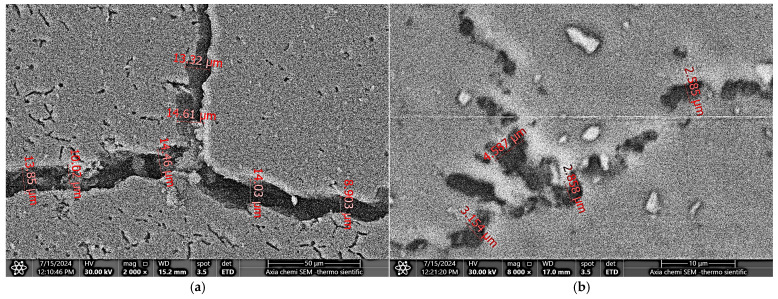
SEM micrographs of the hook cracks of (**a**) a specimen of the conventional process; (**b**) a specimen performed with the inert gas cover.

At corresponding locations in both specimens, a relatively wider and coarser lower crack C1 extends from the interface towards the FBA of the specimen of process A, along with long voids observed in the upper crack C2. In contrast, the specimen from process B exhibited a narrower crack C1′, while shorter voids appeared in the crack C2′. In both welding conditions, kissing bonds can be seen due to the presence of oxide particles in sync with a lower stirring effect of the tool pin a short distance away from the SZs of the specimens, as shown in the magnified views III and IV in Figure 6d,e and Figure 7d,e, respectively. An Axia ChemiSEM scanning electron microscope (SEM), equipped with energy-dispersive spectroscopy (EDS) analysis, was used to examine the hook cracks of the as-welded specimens. Figure 8a,b shows SEM micrographs of the hook cracks of the specimens in Figure 6 and Figure 7, respectively. It can be seen that the crack width ranges between 10 and 14.6 µm in the specimen from the conventional process and between 2.8 and 4.5 µm in the specimen welded under the inert gas cover. These results can be attributed to the greater inclusion of oxide particles in the bond line of the specimens from the conventional welding process compared to those welded with the argon cover. This behavior suggests an improvement in weld quality and the consequent weld strength achieved by employing the inert gas cover during the FSSW process. Rao et al. [9] linked the hook morphology and geometry to the interfacial oxides while studying their effect on the static and fatigue behaviors of magnesium FSSW welds. Similarly, Badarinarayan et al. [21] correlated the hook crack morphology and oxide inclusion to the formation of the partially metallurgical bond area. In a previous study, Klobcar et al. [4] concluded that the bond line oxidation degrades the weld quality, with cracks propagating along the oxide path under static and cyclic loading conditions.

### 3.2. EDS Quantification

As mentioned above, the hook originates from the interfacial crack of the FSSW lap-shear specimen and forms by an array of broken oxide layer particles within the PBA. The characteristics of the hook have a significant impact on the weld quality and strength [7,9,21]. In this study, the hook morphology is deeply analyzed to assess the influence of the interfacial oxide layer on the hook formation, as well as how the use of the inert gas affects the tensile and fatigue behaviors under static and cyclic loading conditions respectively. The same specimens that were examined with an optical microscope in the previous section were re-polished to remove any oxide layer that may have formed due to atmospheric oxygen exposure. The specimens were then etched to reveal the shapes of the hook cracks clearly and stored in plastic containers filled with inert argon gas to prevent the formation of additional oxide layers. This process ensures that only the oxide formed during the welding process is present. Subsequently, the specimens were examined using an energy-dispersive spectroscope (EDS) to analyze the interfacial oxide inclusion in the bond lines. The weight percentage of oxygen was adopted to indicate the oxide content in the tested areas. Although this is not necessarily representative, these percentages can be used comparatively to assess the interfacial oxide inclusion as a procedure specific to this study. The EDS analyses also detected some elemental residuals not found in the base alloy composition, possibly due to contamination or chemical treatment during the metallographic preparations.

**Figure 9:**
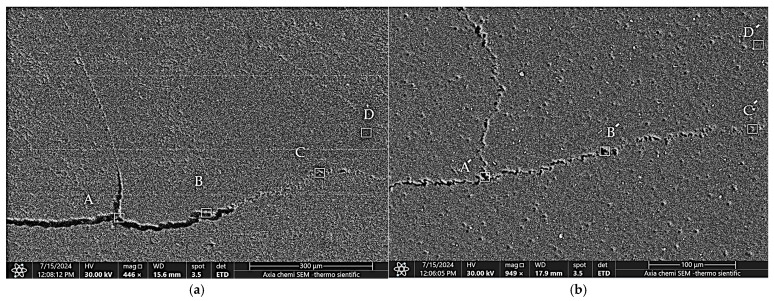
SEM micrographs of EDS analysis for specimens of (**a**) conventional welding process; (**b**) argon-covered welding process.

The purity of the PBA region plays a dominant role in determining the quality and integration of the bond line. Tested areas were chosen that represent critical locations within the interfacial bond line of the weld joint that were most influential in the fusion of the two sheets of the lap-shear specimen. Accordingly, three areas were tested at the beginning, middle, and end of the PBA, in addition to one area in the FBA that was already welded and contained the minimum amount of interfacial oxide particles due to the efficient stirring effect at the periphery of the keyhole. For comparable EDS quantification, the four tested points were selected in corresponding locations in specimens of both welding conditions. Figure 9a,b shows SEM micrographs of the hooks of specimens from A and B sets, respectively. In the figures, four square areas with dimensions of 10 µm × 10 µm A−D and A′−D′ were marked at corresponding locations for the EDS quantification. Table 3 and Table 4 display the EDS spectrum analyses of the interfacial hooks of the specimens from A and B groups shown in Figure 9a,b, respectively. It is important to note that the tables reflect the elemental composition of the hook crack rather than the base metal. In both welding conditions, it was observed that the interfacial oxide content gradually decreased in different areas of the hook cracks as stirring efficiency increased, starting from the hook formation at the bond line at the UBA, passing through the PBA, and approaching the SZ of the weld at the FBA, where the oxide particles were dispersed within the SZ [4]. The EDS quantification results clearly show that the specimen welded with the inert gas cover exhibited lower oxide inclusion in the tested areas A′−D′ of the hook crack compared to the areas A−D in the specimen welded in ambient air. This difference is attributed to the isolation of the joint bond line from the ambient air oxygen during the welding process, which prevents or significantly reduces the formation of the interfacial oxide layer.

### 3.3. Micro-Hardness

**Figure 10:**
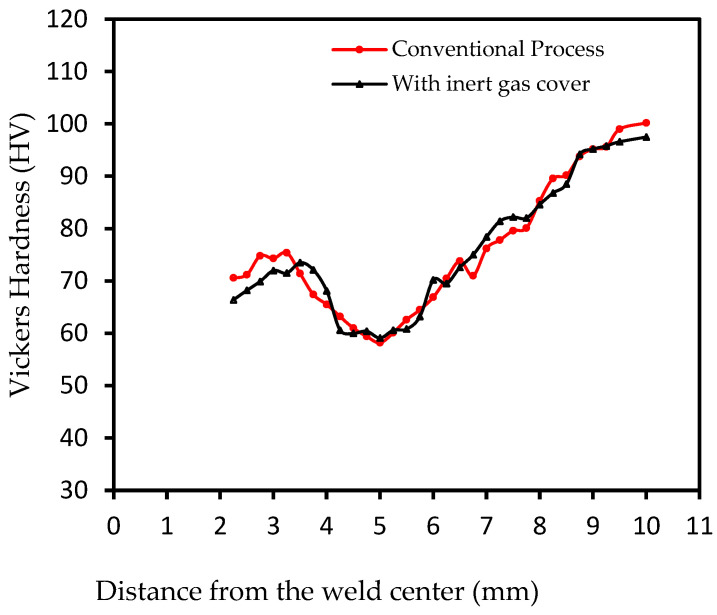
Micro-Vickers hardness for the cross-sections of FSSW specimens from A and B sets.

Prepared specimens from A and B groups were subjected to micro-Vickers hardness testing to assess the influence of the Ar cover on the hardness of the different weld zones within the joints. Figure 10 shows the Vickers hardness distribution for the cross-section of specimens from A and B sets. The figure shows a typical profile for all neighboring weld zones. The figure shows higher hardness values in the SZs due to higher grain refinement achieved by the stirring tool pin effect at full dynamic crystallization at the periphery of the keyhole compared to the neighboring TMAZs. On the other hand, the heat effect resulted in coarse grain size and therefore lower hardness observed at the HAZs compared to the BM areas. The microstructure and how it relates to the hardness values in different zones of the FSSW weld joint have been studied in detail in previous studies conducted by our research team [7,12]. Due to the nearly symmetrical hardness distribution profile with respect to the keyhole centerline, resulting from the symmetrical conditions experienced during the welding process, only one side of the hardness values is represented for the cross-sections of the welded specimens from both welding conditions. Feizollahi et al. [11] concluded different micro-hardness values in corresponding weld zones induced by microstructural differences while investigating the influence of tool shoulder diameter and tool rotational speed on dissimilar FSSW joint strength.

In this work, the specimens exhibited no significant difference in Vickers hardness values. These almost similar hardness profiles are an expected result of the identical welding parameters and tool geometry. This similarity is attributed to the equivalent frictional heat generation and material flow during welding. However, a slight increase in Vickers hardness was observed in the SZ of the specimens from the conventional process compared to those welded with the inert gas cover as a result of the variations in the precipitation environment during the welding process.

### 3.4. Static Strength

During the tensile test, averages of the tensile–shear load (TSL) values of 4055 N and 5750 N were recorded for specimens from groups A and B, respectively. The similarity in hardness between corresponding weld zones for both welding conditions rules out hardness as a factor in explaining the superior TSL values of the specimens welded under inert gas conditions compared to those welded in ambient air. As shown in Figure 6 and Figure 7, the improved morphology, larger FBA, and smaller PBA in the bond line achieved with the inert gas cover likely account for the increase in tensile strength due to the lower inclusion of oxide layer and embedded voids within the weld joint. Previous studies have consistently linked the tensile strength of FSSW-welded joints to the size of the weld nugget area, including [7,12,31,40,41]. The controlled welding atmosphere not only improves the morphology of the bond lines but also enhances material flow and metallurgical integrity within the SZ. By minimizing incomplete bonds and reducing the dispersion of oxide particles, a larger proportion of the fully bonded area is achieved, directly contributing to the increased loads bearing capacity in tensile tests.

### 3.5. Fatigue Behavior Under Cyclic Loading Conditions

#### 3.5.1. Fatigue Strength

**Figure 11:**
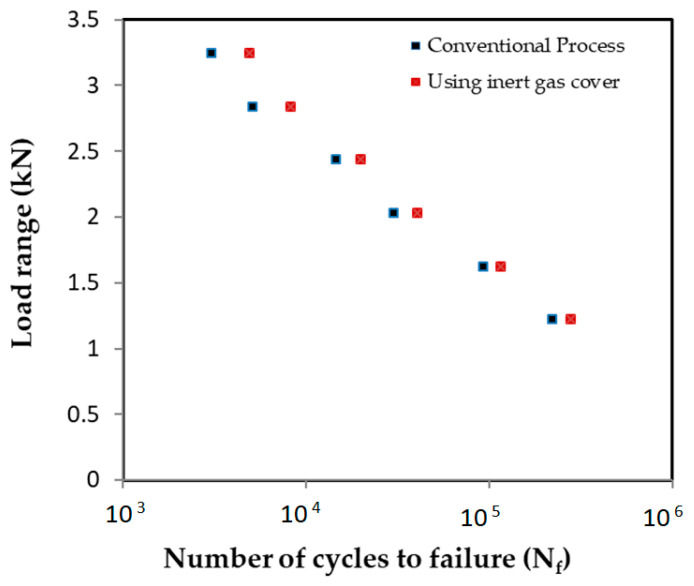
Experimental results of load range as a function of number of cycles to failure.

Figure 11 represents the experimental results of the fatigue test conducted for the conventional and inert gas-covered FSSW processes. The figure illustrates the load levels as a function of fatigue life as the number of cycles to failure (N_f_). The results clearly show that the specimens welded with the argon gas cover exhibited superior fatigue durability for all load levels compared to those welded using the conventional welding process with an improvement varied between 60% and 26% at the higher load level of the low-cycle fatigue and the lower load level of the high-cycle fatigue, respectively. This superiority is clearly seen at low-cycle and high-cycle fatigue regions, indicating a consistent improvement in fatigue performance when the inert gas cover is employed during welding.

Micro-voids which result from oxidation at the bond interface of the welded specimens are typical sites for the initiation of fatigue cracks. These cracks propagate under cyclic loading conditions, finally leading to fatigue failure [1,20,21]. Additionally, the interfacial oxidation results in an extension of the unbonded area (UBA) into the PBA and a wider original interfacial hook crack (see Figure 7e), which negatively affect the static and fatigue strengths of the welds [2,20,21]. In the specimens welded in ambient air, the oxidized interface and the consequent larger PBA represent an ideal path for crack propagation under cyclic loading, as shown in Figure 6 and Figure 8a and Table 5. In contrast, a bond line of higher quality and purity was observed in the specimen performed with the inert gas cover compared to that of the specimen of the conventional FSSW process. Consequently, crack propagation encounters greater resistance due to the improved cohesion in the bond line of the welds protected by the argon cover during the welding process, as shown in Figure 7 and Figure 8b and Table 6.

#### 3.5.2. Failure Mechanism General Overview

The fatigue crack initiates at the original hook crack tip, which originates at the joint interface near the weld nugget. This crack propagates under cyclic loading, favoring the upper sheet of the FSSW lap joint due to the location and direction of the original hook crack tips; as well, the sheet-thinning phenomenon occurs at the upper coupon caused by the shoulder penetration depth during the FSSW process [7,9,20,31].

In low-cycle fatigue, which is associated with relatively high load levels, shear forces play a dominant role in determining the failure mode. The higher force applied to the weld nugget area of the lap-shear specimen leads to significant shear stress, resulting in a shear failure mode during the fatigue test. In contrast, in high-cycle fatigue, which involves lower load levels, the weld nugget experiences lower shear force and, consequently, lower shear stress due to the reduced load range applied to the FSSW lap-shear specimen under cyclic loading conditions.

At lower load levels, weaknesses such as initial crack tips, voids, and oxide inclusions become critical, acting as stress concentration points that favor fatigue crack initiation. However, the areas of the bond line closest to the point bearing the force are most prone to crack initiation and propagation, as regions farther away benefit from greater structural support within the weld joint. As a result, crack initiation, propagation, direction, and inclination follow this effect. With decreasing load levels, fatigue cracks tend to initiate and propagate away from the weld center, passing through the upper sheet thickness, extending around the weld nugget, and often leading to nugget pull-out, circumferential failure, or other modes aside from shear fracture.

**Figure 12:**
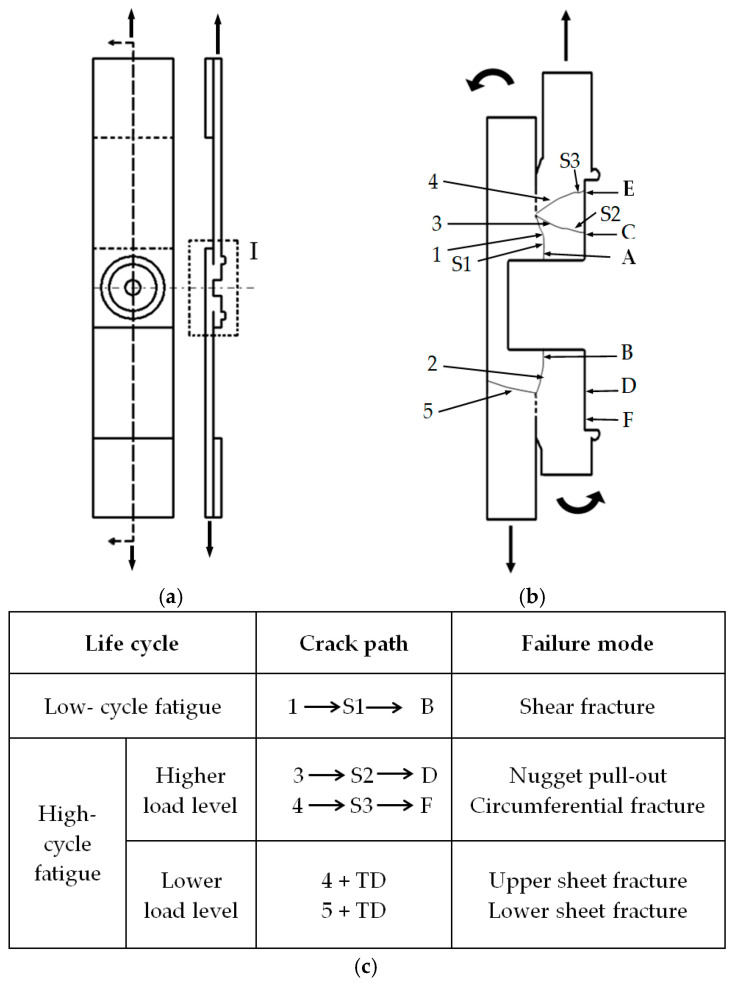
Schematic illustration of the fatigue failure mechanism: (**a**) a top view of the FSSW lap-shear specimen and a cross-section along the symmetry loading axis A-A; (**b**) a magnified view of region I specified in (**a**); and (**c**) two-dimensional representation of the specimen cross-section, including the crack behaviors at different failure modes.

To better understand the failure mechanism of the FSSW lap-shear specimens under cyclic loading conditions, Figure 12 provides a schematic bi-dimensional representation of crack initiation and propagation during the fatigue test, leading to the failure and complete separation of the two coupons of the FSSW specimen. Vertical bold black arrows indicate the cyclic load direction. Figure 12a shows a top view of the FSSW lap-shear specimen and a cross-section along the symmetry axis, while Figure 12b shows a magnified view of the area I of Figure 12a, which represents the weld joint area. In this figure, the dashed lines near the welded area represents the UBA. Multiple cracks may initiate and propagate under cyclic loading, depending on the applied load range, welding conditions, and consequent quality and durability of the weld joint, as well as other favorable stress conditions, but only one crack is ultimately responsible for the failure of the FSSW lap-shear specimen, producing a specific failure mode. Figure 12c summarizes the failure modes in relation to crack propagation paths under low- and high-cycle fatigue conditions.

In low-cycle fatigue, dominant cracks 1 and 2 typically initiate from the original hook crack tips, as shown in Figure 12b. Crack 1 propagates slightly along the interface of the two coupons of the lap-shear specimen under cyclic loading conditions before passing a short distance through the upper sheet thickness until reaching point A. The crack then turns into a shear crack S1, propagating around the periphery of the keyhole. The specimen ultimately fails at point B in a shear fracture mode. This mode of failure is commonly seen in low-cycle fatigue, although it has also been observed in some tests of a lower level of high-cycle fatigue. The upper sheet is bent at the opposite side of the final crack propagation under the test load due to the lack of connection and support with the weld nugget at the fracture side.

At lower load levels in high-cycles fatigue, the crack follows a different path depending on favorable stress conditions. Experimental observations indicated that the fatigue crack initiates a short distance from the weld center. As shown in Figure 12b, crack 3 originates from the crack tip at the interface near the weld nugget. The fatigue crack passes through the upper sheet thickness and turns into a shear crack S2 at position C, propagating around the weld nugget and leading to failure at point D with the nugget pull-out mode. At higher load level, crack 4 follows a similar path, resulting in a circumferential failure mode at point F, as shown in Figure 12b and summarized in Figure 12c. Meanwhile, at a higher-cycle fatigue, cracks 4 and 5 propagate in a transverse direction (TD) due to the favorable stress condition after passing through the thickness of the upper or lower sheet, and the FSSW lap-shear specimen then fails with the upper or the lower sheet transverse fracture, respectively.

In general, dominant cracks initiating from the original interfacial crack tips of the lap-shear specimen are responsible for fatigue failure. The location of fatigue crack initiation and the tendency while propagating through the upper sheet thickness under cyclic loading conditions mainly depend on the applied load level and the joint quality and strength. These factors also determine the initiation location and propagation path of the shear cracks, which ultimately control the failure mode.

Numerous previous experimental studies, including [1,8,9,17,33,35,37,38], have reported different failure modes, depending on the load range. In this study, we summarized the failure mechanism and the different final failure modes of weld joints based on the applied load level, along with the metallurgical justification for these outcomes. Using the inert gas cover during welding had a clear impact on the quality of the weld joint and, consequently, on the fatigue strength, as is detailed below.

#### 3.5.3. Failure Modes

After testing, the completely separated lap-shear specimens from each welding condition were examined to identify the failure modes and to characterize the fracture surfaces of the top and bottom sheets. The observed failure modes varied, depending on the load levels, as documented through visual inspections during the fatigue tests.

In this study, a life cycle value of 1.4 × 10^4^ was considered as the transition point between low-cycle and high-cycle fatigue. As such, life cycles ranging from 1 × 10^3^ to 1.4 × 10^4^ were considered as low-cycle fatigue, while life cycles from 1.4 × 10^4^ to 3 × 10^5^ were considered as high-cycle fatigue. It is important to note that this criterion was considered only for the purpose of this study to clarify and represent the observed failure modes.

Visual observations revealed that failure modes varied from shear fracture and nugget pull-out modes at low-cycle fatigue to circumferential and transverse fracture modes at the high-cycle fatigue, depending on the load levels. However, a mixed failure mode, combining shear fracture and nugget pull-out, was also observed at low-cycle fatigue. This mixed failure mode was previously reported by Song et al. [42] in their study on the mechanical properties of FSSW lap joints of AA6061-T6 aluminum alloy. Table 5 and Table 6 summarize the load levels, corresponding load values, macrographs of the fracture surfaces of the upper and lower coupons, and observed failure modes for the FSSW lap-shear specimens welded in ambient air and with the inert gas cover, respectively.

#### 3.5.4. Failure Modes Under Low-Cycle Fatigue Conditions

**Figure 13:**
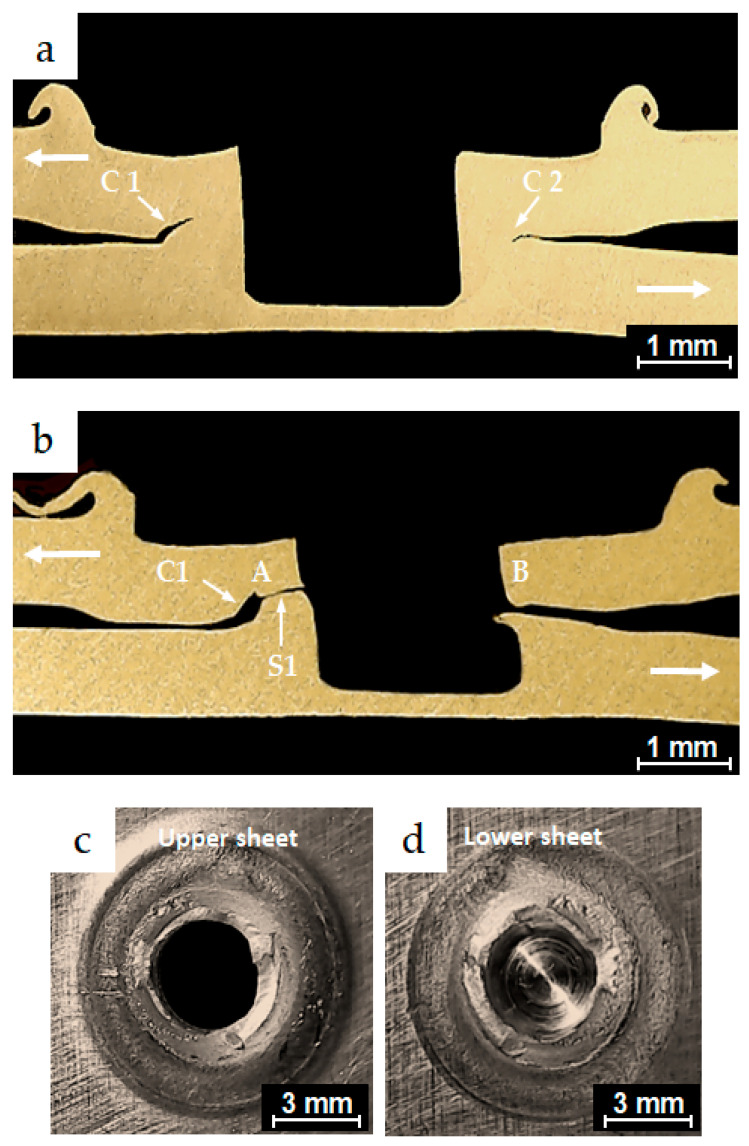
Optical micrographs of the cross-sections along the planes of symmetry of (**a**) a partially failed conventional FSSW specimen, which exhibited fatigue life of 2.3 × 10^3^ cycles under a test load level of 3.244 kN; (**b**) a failed conventional FSSW weld, which exhibited fatigue life of 3.06 × 10^3^ cycles under a test load level of 3.244 kN; (**c**,**d**) the upper and the lower sheets of the failed specimen in (**b**), respectively.

**Figure 14:**
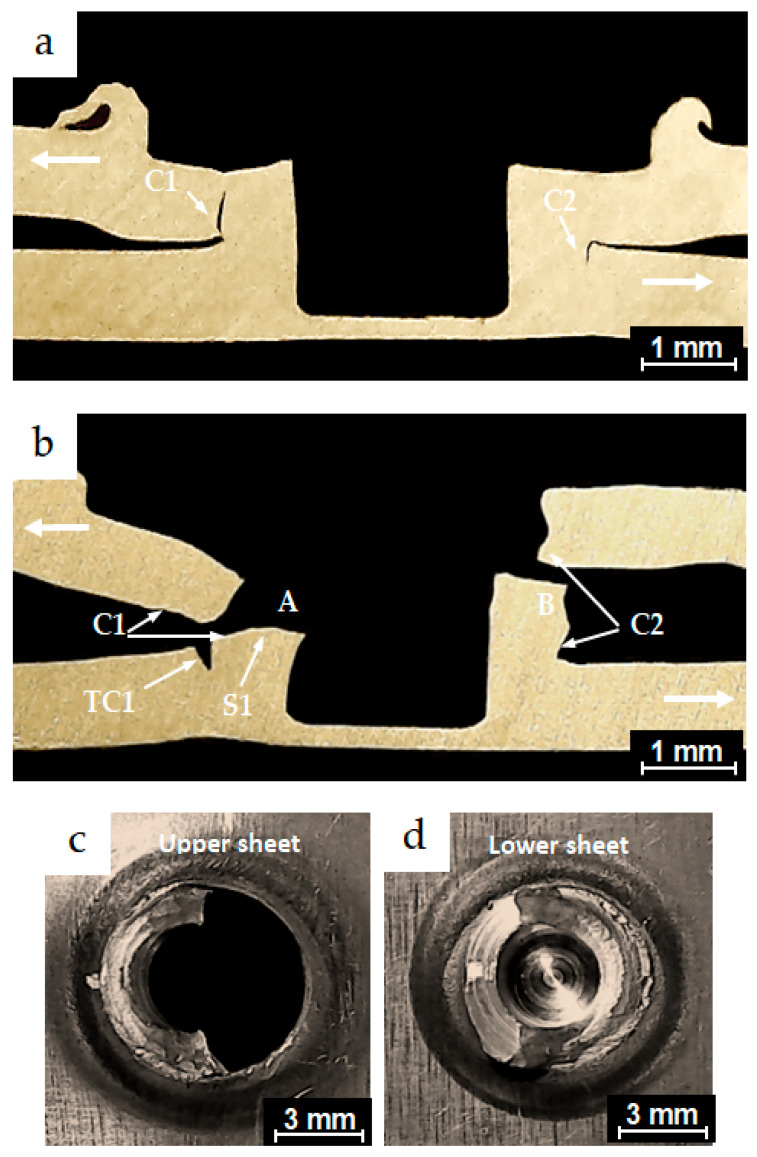
Optical micrographs of the cross-sections along the planes of symmetry of (**a**) a partially failed FSSW specimen performed with the inert gas cover, which exhibited fatigue life of 6.5 × 10^3^ cycles under a test load level of 2.838 kN; (**b**) a failed FSSW specimen performed with the inert gas cover, which exhibited fatigue life of 8.2 × 10^3^ cycles under a test load level of 2.838 kN; (**c**) and (**d**) the upper and the lower sheets of the failed specimen in (**b**), respectively.

The micrographs in Figure 13a,b represent cross-sections along the planes of symmetry for partially and completely failed specimens from the conventional FSSW process, which exhibited fatigue lives of 2.3 × 10^3^ and 3.06 × 10^3^ cycles, respectively. Both specimens were tested under the same load of 3.244 kN. The cyclic test load directions applied to the specimen legs are indicated by the bold white arrows. The figure shows dominant cracks, C1 and C2, which initiated from the original hook crack tips of the FSSW specimens. Under cyclic loading conditions, crack C1 propagated slightly along the interface of the two coupons of the lap-shear specimen before advancing through the thickness of the upper sheet, reaching position A. C1 then turned into a shear crack S1, which propagated around the periphery of the keyhole. The specimen finally failed at position B in a shear fracture mode, as shown in the separate upper and lower coupons of the lap-shear specimen in Figure 13c,d, respectively. Similarly, the micrographs shown in Figure 14a,b represent cross-sections along the planes of symmetry for partially and completely failed specimens from the FSSW process performed with the inert gas cover. These specimens exhibited fatigue lives of 6.5 × 10^3^ and 8.2 × 10^3^, respectively, and were tested under the same load of 2.838 kN. Dominant cracks C1 and C2 initiated from the original interfacial crack tip. Under cyclic loading conditions, crack C1 propagated a short distance along the interface of the two coupons of the lap-shear specimen, before converting into a shear crack S1 that passed through the SZ of the weld nugget and slightly through the thickness of the upper sheet. Crack S1 initially propagated at point A, then progressed around the periphery of the keyhole and gradually around the SZ of the joint. These specimens exhibited a higher fatigue life within the low-cycle fatigue range compared to those from the conventional welding process, and finally failed at point B in a mixed failure mode of shear and nugget pull-out fracture. This failure mode can be seen in the separate upper and lower coupons in Figure 14c,d, respectively. Additionally, transverse crack TC1 was observed initiating from the original joint interface and propagating a short distance through the lower sheet thickness, although it did not play a significant role in causing the failure. These are common typical crack behaviors observed at the low-cycle fatigue conditions in previous studies [37,38].

As summarized in Table 5 and Table 6, under low-cycle fatigue conditions, the conventional FSSW welds predominantly showed a shear fracture failure mode, while specimens welded with the inert gas cover exhibited both shear fracture and a mixed mode of shear fracture and nugget pull-out failure. These failure modes have been commonly observed in low-cycle fatigue conditions in friction stir spot welds in previous studies [1,8,33,37,38].

#### 3.5.5. Failure Modes Under High-Cycle Fatigue Conditions

**Figure 15:**
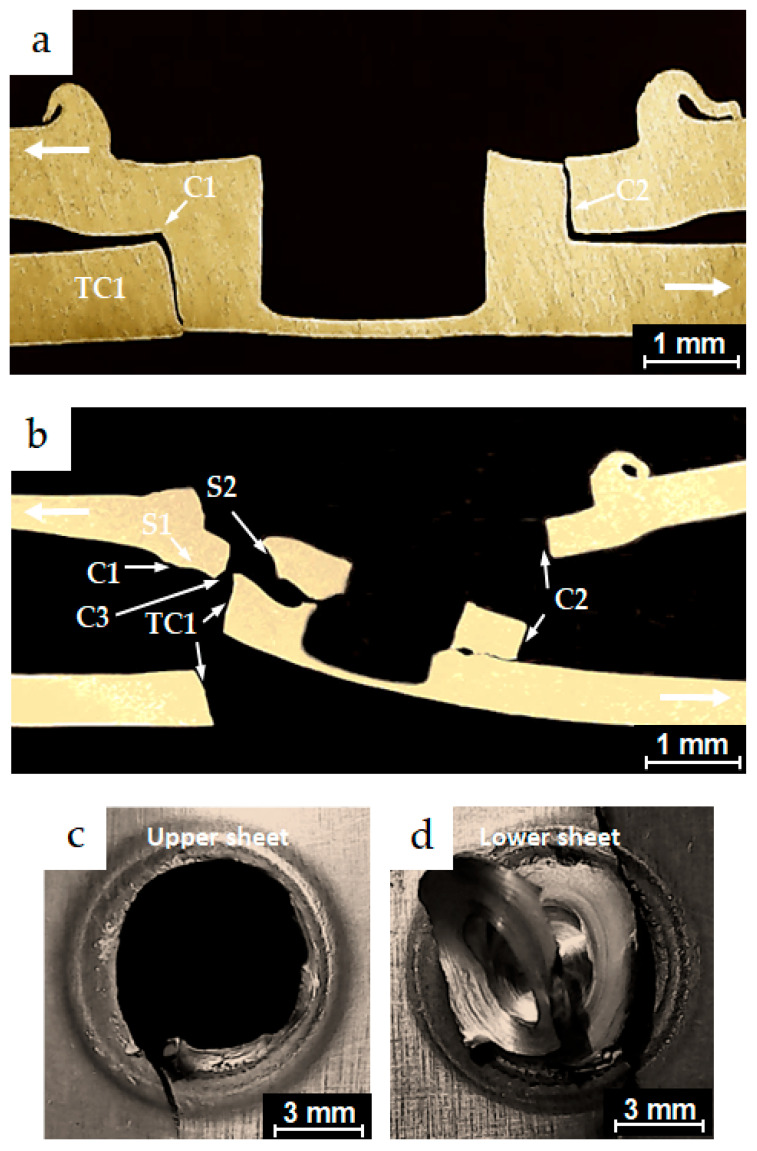
Optical micrographs of the cross-sections along the planes of symmetry of (**a**) a partially failed conventional FSSW specimen, which exhibited fatigue life of 6.8 × 10^4^ cycles under a test load level of 1.622 kN; (**b**) a failed conventional FSSW weld, which exhibited fatigue life of 9.3 × 10^4^ cycles under a test load level of 1.622 kN; (**c**) and (**d**) the upper and the lower sheets of the failed specimen in (**b**), respectively.

The micrographs shown in Figure 15a,b represent cross-sections along the planes of symmetry for partially and completely failed specimens from the conventional FSSW process, which exhibited fatigue lives of 6.8 × 10^4^ and 9.3 × 10^4^, respectively. Both specimens were tested under the same load of 1.622 kN.

During the lower range of high-cycle fatigue, in addition to the two dominant cracks C1 and C2 observed under low-cycle fatigue conditions, an additional crack C3 was observed initiating from the joint interface and propagating through the upper sheet thickness. A transverse crack TC1 also initiated from the original interfacial crack tip of the lap-shear specimen and propagated through the lower sheet thickness, as shown in Figure 15b. Under cyclic loading conditions, dominant crack C1 propagated slightly along the weld interface before extending into the upper sheet thickness, then turned into a shear crack S1, propagating around the periphery of the keyhole without causing the final failure. Crack C3 then converted into another shear crack S2, initially propagating around the periphery of the weld nugget and gradually around the shoulder indentation, leading to final failure. The specimen failed in a mixed mode of nugget pull-out and circumferential failure, as shown in the separate upper and lower coupons of the lap-shear specimen in Figure 15c,d, respectively.

**Figure 16:**
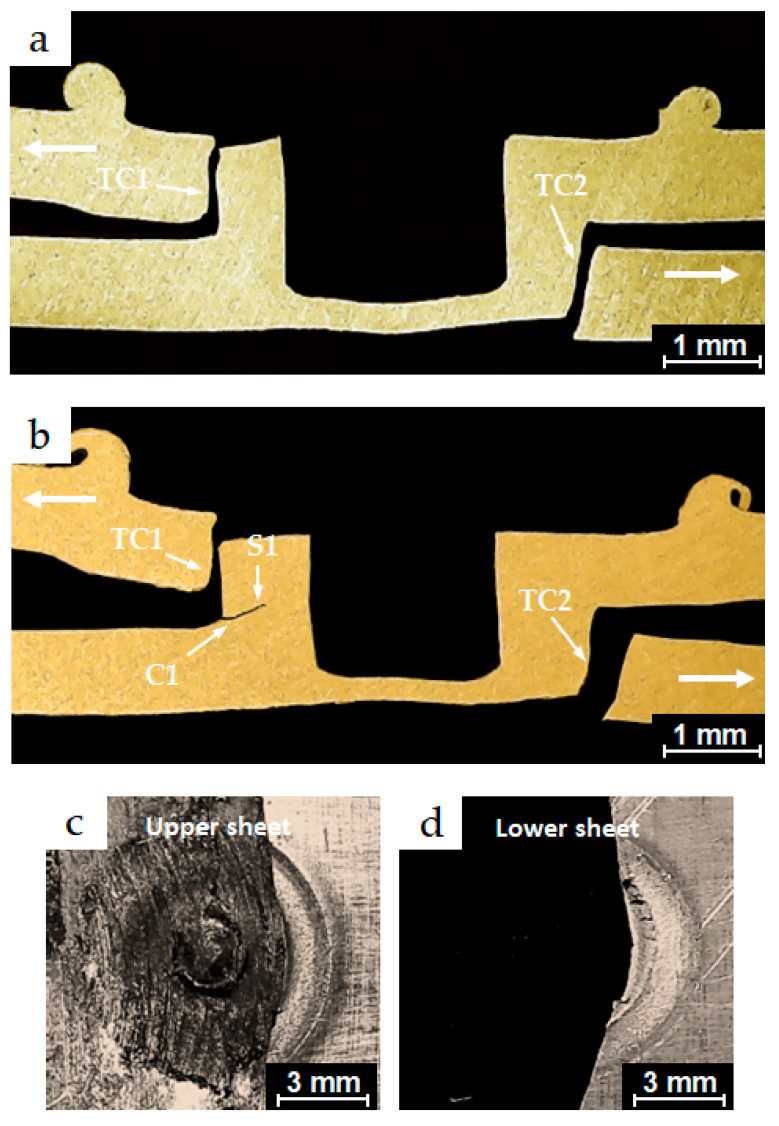
Optical micrographs of the cross-sections along the planes of symmetry of (**a**) a partially failed FSSW specimen performed with the inert gas cover, which exhibited fatigue life of 8.6 × 10^4^ cycles under a test load level of 1.622 kN; (**b**) a failed FSSW specimen performed with the inert gas cover, which exhibited fatigue life of 2.8 × 10^5^ cycles under a test load level of 1.216 kN; (**c**) and (**d**) the upper and the lower sheets of the failed specimen in (**b**), respectively.

The micrographs shown in Figure 16a,b show cross-sections along the planes of symmetry for partially and completely failed specimens from the FSSW process welded with inert gas cover. These specimens exhibited fatigue lives of 8.6 × 10^4^ and 2.8 × 10^5^ cycles, tested under 1.622 kN and 1.216 kN, respectively. At a higher-cycle fatigue, two transverse cracks TC1 and TC2 were observed initiating from the original interfacial crack tips and propagating transversely through the upper and lower sheet thicknesses by the favorable stress conditions. The specimen ultimately failed in the lower sheet transverse failure mode, as can be seen in the separate upper and lower coupons in Figure 16c,d, respectively. Additionally, crack C1 initiated from the original interfacial crack tip, propagated a short distance along the interface of the two sheets and through the upper sheet thickness, and then turned into a shear crack S1, but this crack did not significantly contribute to the final failure of the joint, as shown in Figure 16b.

As summarized in Table 5 and Table 6, under high-cycle fatigue conditions, the failed FSSW welds from both welding conditions showed different failure modes, including nugget pull-out, circumferential failure, upper sheet transverse failure, and lower sheet transverse failure. These failure modes have been commonly observed in high-cycle fatigue conditions in friction stir spot welds, as reported in previous studies [1,8,33,37,38].

## 4. Conclusions

This study investigated the effect of using an inert gas cover of argon on the static and fatigue behavior of AA6061-T6 aluminum alloy friction stir spot welds (FSSW) in a lap-shear configuration. Based on the experimental methodologies and observations during welding, specimen testing, and micrograph analysis, the following key findings were reported:

(1) Optical and scanning electron micrographs revealed that the FSSW specimens performed with the inert gas cover exhibited a significant enlargement in the fully bonded area (FBA) by approximately 33%, along with a reduced partially bonded area (PBA) by 33%, in addition to a lower width range of hook cracks compared to those of the conventional welding process;
(2) The energy-dispersive spectroscopy (EDS) analysis showed a lower oxide inclusion, in terms of oxygen weight percentages (wt.%), in various hook crack regions within the joint bond line when the inert argon gas cover was used during the FSSW welding process. A reduction of 41% and 32% was achieved in the unbounded area (UBA) and the PBA, respectively, while no considerable oxide inclusion formed in the FBA due to the efficient stirring effect at the periphery of the welding tool pin;
(3) Micro-hardness tests showed no significant differences in hardness distribution across the corresponding weld zones of both welding processes, attributed to the same material, identical welding parameters, and consistent tool geometry;
(4) Specimens welded with the inert gas cover exhibited a 14% increase in tensile strength compared to those produced using the conventional process;
(5) The fatigue life of the specimens welded with the inert gas cover was longer at all load ranges in both low-cycle and high-cycle fatigue conditions compared to the conventional process, with an improvement that varied between 60% and 26% at the higher load level of the low-cycle fatigue and the lower load level of the high-cycle fatigue, respectively;
(6) Fatigue failure was primarily caused by dominant cracks initiating at the original interfacial crack tips of the FSSW lap-shear specimens and propagating under cyclic loading;
(7) The fatigue crack initiation and tendency during propagating through the upper sheet thickness under cyclic loading conditions mainly depend on the applied load level, the hook crack morphology, and the bond line quality and purity. Consequently, the shear crack initiation site, the propagation path, and the mode of failure were suggested;
(8) Under low-cycle fatigue conditions, the failed FSSW welds from the conventional process showed a shear fracture failure mode, whereas those welded with the inert gas cover specimens performed with the inert gas cover exhibited both shear fracture and a mixed mode of shear fracture and nugget pull-out failure modes. On the other hand, under high-cycle fatigue conditions, the failed welds from both processes showed different failure modes, including nugget pull-out, circumferential failure, upper sheet transverse failure, and lower sheet transverse failure.

## Figures and Tables

**Table 1:** Chemical composition analysis (Wt.%) of AA6061-T6 aluminum alloy [35,36].

Element	Si	Fe	Cu	Mn	Mg	Zn	Cr	Ti	Al
**wt (%)**	0.54	0.46	0.32	0.07	0.69	0.009	0.20	0.05	Balance

**Table 2:** Mechanical properties of AA6061-T6 aluminum alloy [3,12].

0.2% Yield Strength (MPa)	Tensile Strength (MPa)	Shear Strength (MPa)	Hardness (HV)
276	310	207	107

**Table 3:** EDS analysis (wt.%) of areas A−D marked in Figure 9a.

Spectrum	C	O	Mg	Al	Si	Fe	Cu	Cr	Cl	F	Mn	Total
**A**	22.2	11.3	0.4	61.0	4.2	0.5	0.2	0.2	−	−	−	100.0
**B**	17.9	8.6	0.5	67.7	2.8	1.4	−	−	0.6	0.6	−	100.0
**C**	18.0	7.2	0.4	73.3	−	0.7	−	−	−	−	0.3	100.0
**D**	15.5	1.9	0.4	81.0	0.5	0.7	−	−	−	−	−	100.0

**Table 4:** EDS analysis (wt.%) of areas A′−D′ marked in Figure 9b.

Spectrum	C	O	Mg	Al	Si	Fe	Cu	Cr	Cl	Total
**A**′	18.8	6.6	0.4	72.5	0.5	0.9	0.3	−	−	100.0
**B**′	18.0	5.8	0.5	74.4	0.3	0.7	−	0.2	−	100.0
**C**′	17.2	4.9	0.5	76.4	0.4	0.6	−	−	−	100.0
**D**′	16.5	1.8	0.5	80.2	−	0.7	−	−	0.2	100.0

**Table 5:** Failure modes and fracture surfaces at different load levels of specimens welded with the conventional welding process.

Load Level (%)	Load Value (kN)	Fracture Surfaces		Failure Mode
		Upper Sheet Bottom	Lower Sheet Top	
80	3.244	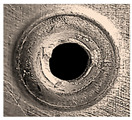	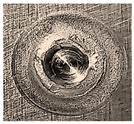	Shear fracture
70	2.838	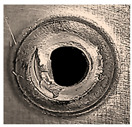	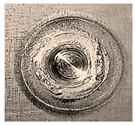	Shear fracture
60	2.433	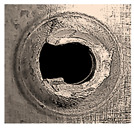	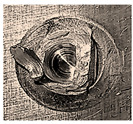	Shear fracture + Nugget pull-out (Mixed mode)
50	2.027	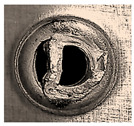	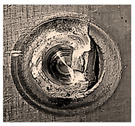	Nugget pull-out
40	1.622	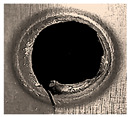	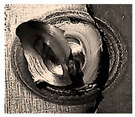	Circumferential failure mode
30	1.216	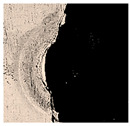	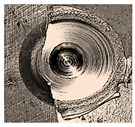	Upper sheet transverse fracture

**Table 6:** Failure modes and fracture surfaces at different load level of specimens welded with the inert gas cover.

Load Level (%)	Load Value (kN)	Fracture Surfaces		Failure Mode
		Upper Sheet Bottom	Lower Sheet Top	
80	3.244	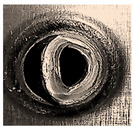	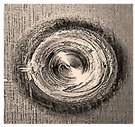	Shear fracture
70	2.838	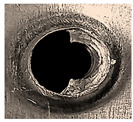	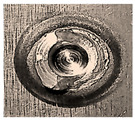	Shear fracture + Nugget pull-out (Mixed mode)
60	2.433	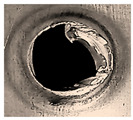	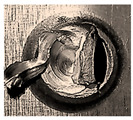	Nugget pull-out
50	2.027	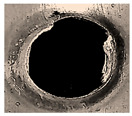	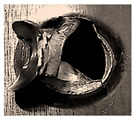	Circumferential fracture
40	1.622	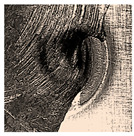	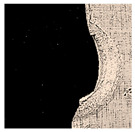	Lower sheet transverse fracture
30	1.216	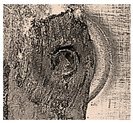	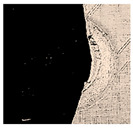	Lower sheet transverse fracture

## Data Availability

The original contributions presented in this study are included in the article. Further inquiries can be directed to the corresponding author.
